# A high-level 3D visualization API for Java and ImageJ

**DOI:** 10.1186/1471-2105-11-274

**Published:** 2010-05-21

**Authors:** Benjamin Schmid, Johannes Schindelin, Albert Cardona, Mark Longair, Martin Heisenberg

**Affiliations:** 1 Department of Neurobiology and Genetics, Biocenter, University of Würzburg, Am Hubland, Würzburg, Germany; 2 Max Planck Institute of Molecular Cell Biology and Genetics, Pfotenhauerstrasse 108, Dresden, Germany; 3 Institute of Neuroinformatics, Uni/ETH Zürich, Winterthurerstrasse 190, Zürich, Switzerland; 4 School of Informatics, University of Edinburgh,10 Crichton Street, Edinburgh, UK

## Abstract

**Background:**

Current imaging methods such as Magnetic Resonance Imaging (MRI), Confocal microscopy, Electron Microscopy (EM) or Selective Plane Illumination Microscopy (SPIM) yield three-dimensional (3D) data sets in need of appropriate computational methods for their analysis. The reconstruction, segmentation and registration are best approached from the 3D representation of the data set.

**Results:**

Here we present a platform-independent framework based on Java and Java 3D for accelerated rendering of biological images. Our framework is seamlessly integrated into ImageJ, a free image processing package with a vast collection of community-developed biological image analysis tools. Our framework enriches the ImageJ software libraries with methods that greatly reduce the complexity of developing image analysis tools in an interactive 3D visualization environment. In particular, we provide high-level access to volume rendering, volume editing, surface extraction, and image annotation. The ability to rely on a library that removes the low-level details enables concentrating software development efforts on the algorithm implementation parts.

**Conclusions:**

Our framework enables biomedical image software development to be built with 3D visualization capabilities with very little effort. We offer the source code and convenient binary packages along with extensive documentation at http://3dviewer.neurofly.de.

## Background

Life sciences are experiencing an increasing demand for scientific image processing. Images are the primary data of developmental and cell biology. The number of images is exploding with the availability of high-throughput and high-resolution technologies. The acquisition of large three-dimensional (3D) data sets, often as time series (4D), has become the new standard.

The first step in the analysis of biological image data is its visual inspection. In addition to the general requirement for visualization, the unique characteristics of each data set may demand specialized analysis. The development of novel analytical tools is greatly facilitated by the existence of well-documented software libraries. These libraries must provide (1) means to load and save any of the large diversity of image file formats; (2) implementations for computer vision algorithms; and (3) graphical user interfaces for data access by a human operator.

We have identified a lack of accessible 3D/4D visualization software libraries for biological image processing. Numerous image processing packages exist, either commercial (Amira, Visage Imaging; MeVisLab, Mevis; Imaris, BitPlane; Volocity, PerkinElmer) or open source (VOXX, [1]; VTK and VTK-based applications such as Slicer3D, BioImageXD, and V3D [2]; UCSF Chimera [3]; VolumeJ [4] and Volume Viewer [5]). These packages offer excellent solutions for the specific problems they were designed to solve. While end-users benefit from well-documented, special-purpose commercial applications, the development of custom analytical tools is better handled by open source packages. The application programming interfaces of existing packages range from the non-existent for most closed commercial solutions, to the very detailed and comprehensive open source VTK environment.

We have created a software library for 3D/4D visualization, with functions for surface extraction, volume rendering and interactive volume editing. Our library removes all the complexity of creating and interacting with image volumes and meshes in a 3D environment. We have designed our library to enrich the core functionality of ImageJ (and its descendant Fiji [6]), an open source image processing application. Via ImageJ, our library has access to hundreds of biological image file formats. Over the years, the scientific community has contributed a very large number of ImageJ extensions, known as plugins, which provide readily accessible implementations of numerous computer vision algorithms. With our library, we empower the ImageJ scientific community to rapidly implement custom analytical tools for 3D/4D data sets, with a minimal investment of time and resources in handling the complex details of a hardware-accelerated 3D environment. This reduction in the difficulty of visualizing 3D information commoditizes the usage of a 3D scene. For example, our library enables software developers to visually assess the correctness of individual algorithmic steps, such as the 3D shape of a mesh deformation. The simplicity of our library is in stark contrast to existing libraries such as VTK, which require detailed knowledge of the underlying data structures.

An example application in need of effective 3D visualization is the image volume reconstruction from electron tomography data as provided by TomoJ [7], an ImageJ plugin. Our library complements TomoJ, enabling convenient 3D analysis of the results without the need of external software such as Chimera [3]. Many other plugins for image registration or object segmentation are similarly using our library for integrated and interactive 3D visualization.

## Implementation

**Figure 1:**
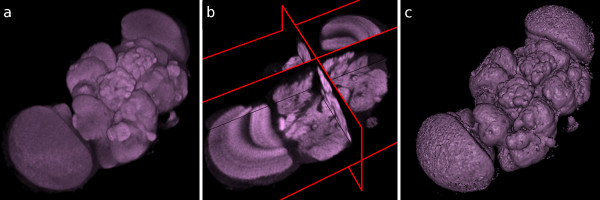
**Three display modes supported by the framework**. An example image volume containing an adult *Drosophila *brain, shown (a) as a volume rendering, (b) as orthoslices and (c) as an isosurface of its external contour.

Our software library implements the concept of three-dimensional scene ("3D scene") for the interactive visualization of biomedical image data (Figure 1). The 3D scene is a virtual 3D space in which any number of entities are hosted. These entities are volume renderings, isosurfaces and orthoslice sets. Volume renderings are representations of voxel data viewed from arbitrary angles, with transparency determined by the intensity values. Isosurfaces are meshes of triangles described by a list of vertices. Orthoslices are orthogonal planes that cut through an image volume. Each of these entities, or objects, has a number of properties, such as transparency, color, and a unique name. The 3D scene that hosts all objects has the following properties: a zooming level, an origin of viewer coordinates, and a scene background.

For efficient rendering in computer graphics, we chose Java 3D: a low-level hardware-accelerated software library. Java 3D has the further advantage of being implemented for Java, thus enabling Java applications like ImageJ to interoperate with the graphics card of a computer, via either OpenGL or DirectX low-level native layer. Java 3D provides a fine-grained representation of a virtual scene as a directed acyclic graph [8]. Operations on any node of the graph affect its entire subtree. In practice, this means that, for example, zooming in and out of a scene is expressed as a scaling transformation in a low-order node. High-order nodes encapsulate images and meshes.

**Figure 2:**
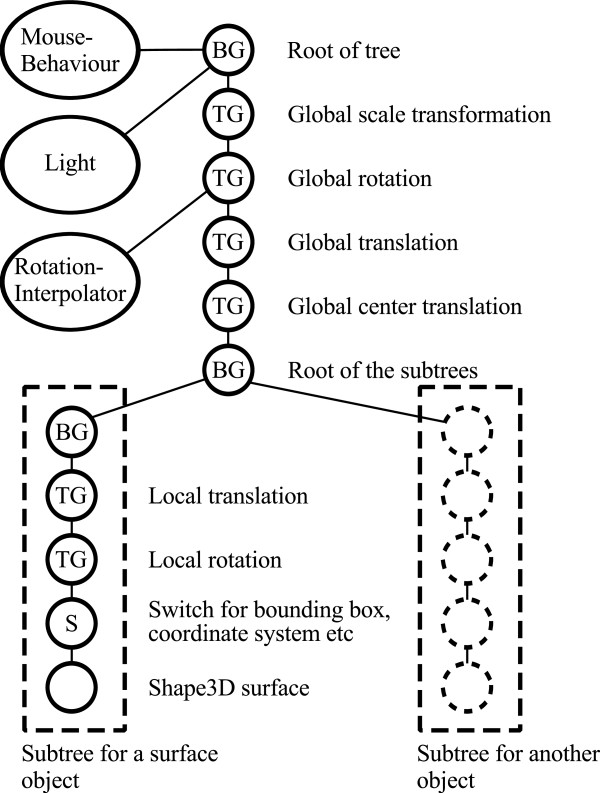
**Scene graph structure of the framework components**. The Java 3D library offers a set of classes that are organized in a directed acyclic graph. The relationships of the nodes in the graph determine how they are rendered in the 3D scene. Our framework, built on top of Java 3D, consists of a chain of global TransformGroup (TG) nodes that represent the view's zoom, rotation and panning (*top*), and which affect the rendering of all subtree nodes. Subtree nodes contain the image volumes. Other nodes include BranchGroup (BG) nodes, capable of holding several subtrees (each representing the internal state and data of an image volume or mesh; *lower right*). Switch (S) nodes are immediate parents of data nodes, and are used to toggle their visibility state.

A key feature of our library is to substantially simplify the usage of Java 3D. We define our 3D scene in terms of Java 3D nodes (Figure 2). We provide straightforward means to instantiate a new interactive 3D scene and to add objects to it. In the following, we describe the structure of the scene graph as implemented in our library.

### Introduction to Java 3D library nomenclature

Java 3D provides a collection of object templates, referred to as classes, each of which represents a node of the scene graph. The most relevant classes are:

• BranchGroup: A node capable of being the root of several subtrees.

• TransformGroup: A node that transforms the spatial representation of its enclosed subtree.

• Switch: A node to toggle on and off the visibility of its subtree nodes.

• Shape3D: A node representing a displayable object. The visualization of a Shape3D is specified by its Appearance and Geometry.

• Geometry: Defines the geometry of the corresponding Shape3D, i.e. its vertex coordinates.

• Appearance: Defines several attributes of a Shape3D regarding its visualization, such as color, transparency and texture.

### Core classes of the library

**Figure 3:**
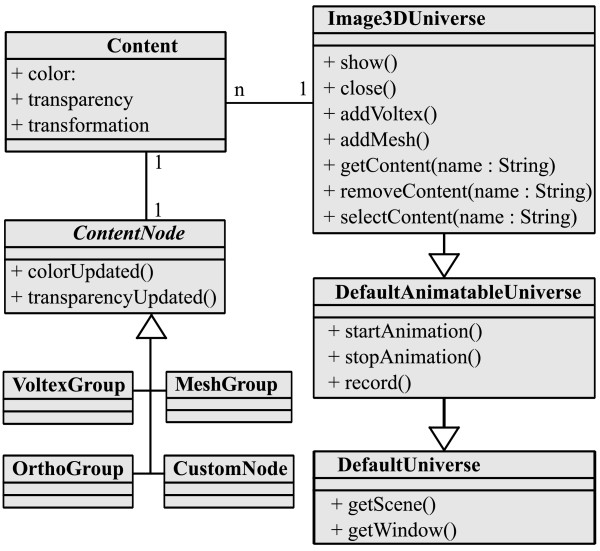
**Class diagram of the framework**. A schematic diagram of the principal classes in our framework, illustrating their relationships. Note that only two classes (Image3DUniverse and Content) are essential for instantiating a new 3D scene with objects in it. The Image3DUniverse class represents the 3D scene, ready for user interaction. The Content class wraps data types like image volumes and meshes, each with a set of properties such as color, transparency and a local transformation. *arrows *indicate class inheritance. *n *indicates that the class contains numerous references to instances of the other class. *1 *indicates that the class contains one reference to the other class.

Our library is composed of about ten different modules. We review here the core module, named ij3d, which interacts with all other service modules. An overview of the module structures and their member classes is shown in Figure 3.

The core ij3d module contains two principal classes, namely Content and Image3DUniverse. The Content class is a high-level representation of a single element in the 3D scene, such as a volume rendering or an isosurface. The Image3DUniverse (1) represents the 3D scene itself; (2) provides simplified access for adding, editing, selecting and removing Content instances to and from the scene, and (3) controls the view transform that represents zooming and panning. Via its superclass DefaultAnimatableUniverse, the Image3DUniverse also provides methods for 3D animation and recording movies.

In addition to data elements, the 3D scene can also contain analytical elements such as annotations in the form of named landmark points. These are added either interactively, or programmatically by accessing a Content instance.

As mentioned above, all elements of the 3D scene are related in a graph structure. Our constructed Java 3D graph links image objects (as Content instances) by wrapping them in ContentNode objects. The latter extend the functionality of basic Java 3D BranchGroup class, to serve as high-level scene elements. The ContentNode class is abstract; the four classes VoltexGroup, MeshGroup, OrthoGroup and CustomNode respectively represent volume renderings, surface renderings, orthoslices and custom geometries.

In summary, our library provides the means to instantiate a 3D scene with a simple user interface for interactions such as zooming, panning, editing objects and recording movies. Programmatically, the task of adding content to the scene has been reduced to a handful of lines of code (see listing 1), which is in stark contrast with the hundreds of lines of code required to achieve the same result using Java 3D directly.

## Results

### Features

We outline the features of our 3D visualization framework. We then describe its usage via both a graphical user interface (GUI) (for end-users) and an application programming interface (API) (for programmers).

#### The 3D scene

The 3D scene is a virtual 3D space in which image volumes and meshes are displayed. Biological image volumes in the form of stacks of 2D images are shown within the 3D space in one of three ways: as a volume rendering, a mesh, or an orthoslice set. Volume rendering [9] is a technique for displaying image volumes directly. An arbitrarily-oriented image volume is projected to the screen with a transfer function such that dark pixels are more transparent than bright pixels. Meshes are constructed by applying the marching cubes algorithm [10] to image volumes to find a surface that encloses all pixels above a desired threshold value. Finally, orthoslices represent three perpendicular and adjustable planes that cut through the volume. An example of each type is shown in Figure 1. The 3D scene is capable of simultaneously hosting multiple image volumes, meshes and orthoslice sets. Each represented image volume has several adjustable attributes such as color, transparency and a local 3D transformation.

#### The toolbar

ImageJ's toolbar offers a collection of region of interest (ROI) tools. Closed ROIs, like rectangles, ellipses and polylines are used for interacting with image volumes (see "Volume editing" below). The point tool adds 3D landmarks, which are represented as small spheres.

#### Volume editing

**Figure 4:**
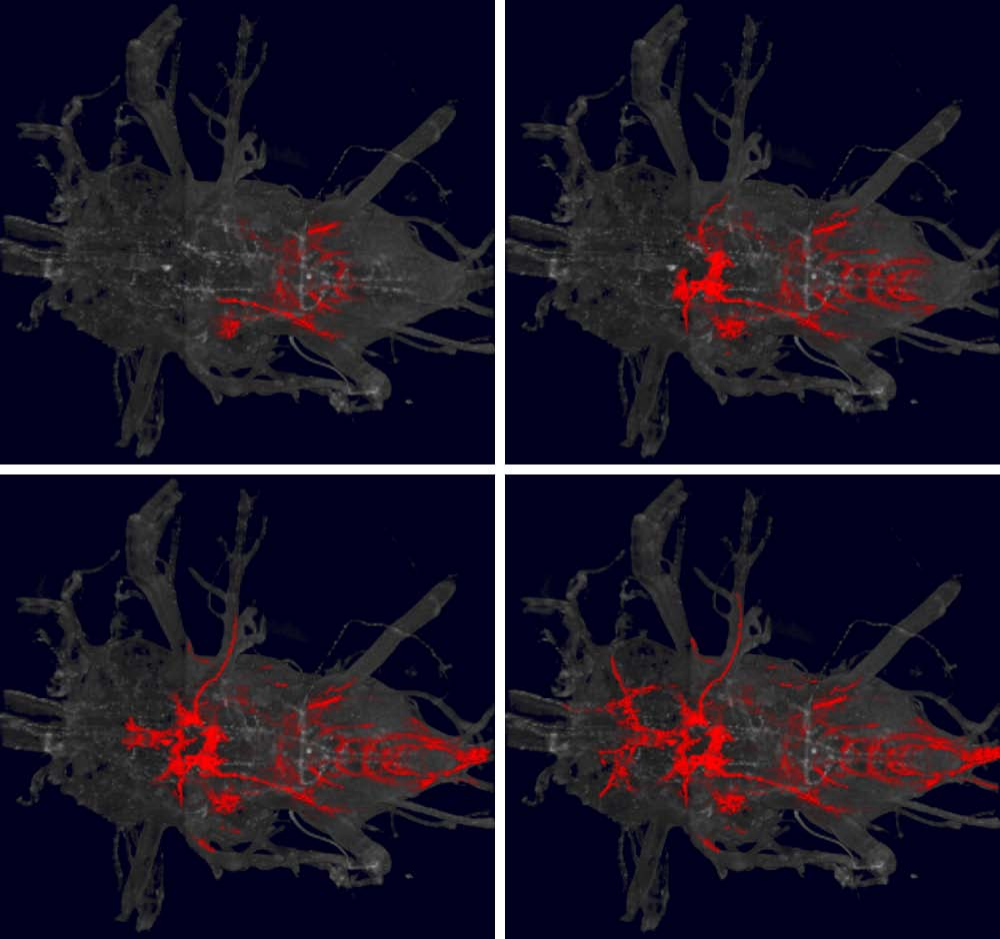
**Animated simulation of dendritic growth**. Four frames of a time sequence, depicting a simulation of dendritic growth in the *Drosophila *thorax. *Gray background*, a volume rendering of the thorax. Dendrites are shown in *red*. Each time frame was generated by directly editing voxels in an image volume, which automatically updates the 3D scene and renders the frame. A complete movie is provided by Additional file 2.

Programmatically, our library provides access to the values of all voxels in an image volume. Changes to voxel values are propagated to the screen. We use this feature for simulating the dendritic growth over time in the thorax of a fruit fly Drosophila (Figure 4). More material about this aspect is available in form of source code (Additional file 1, section 2) and a movie (Additional file 2).

**Figure 5:**
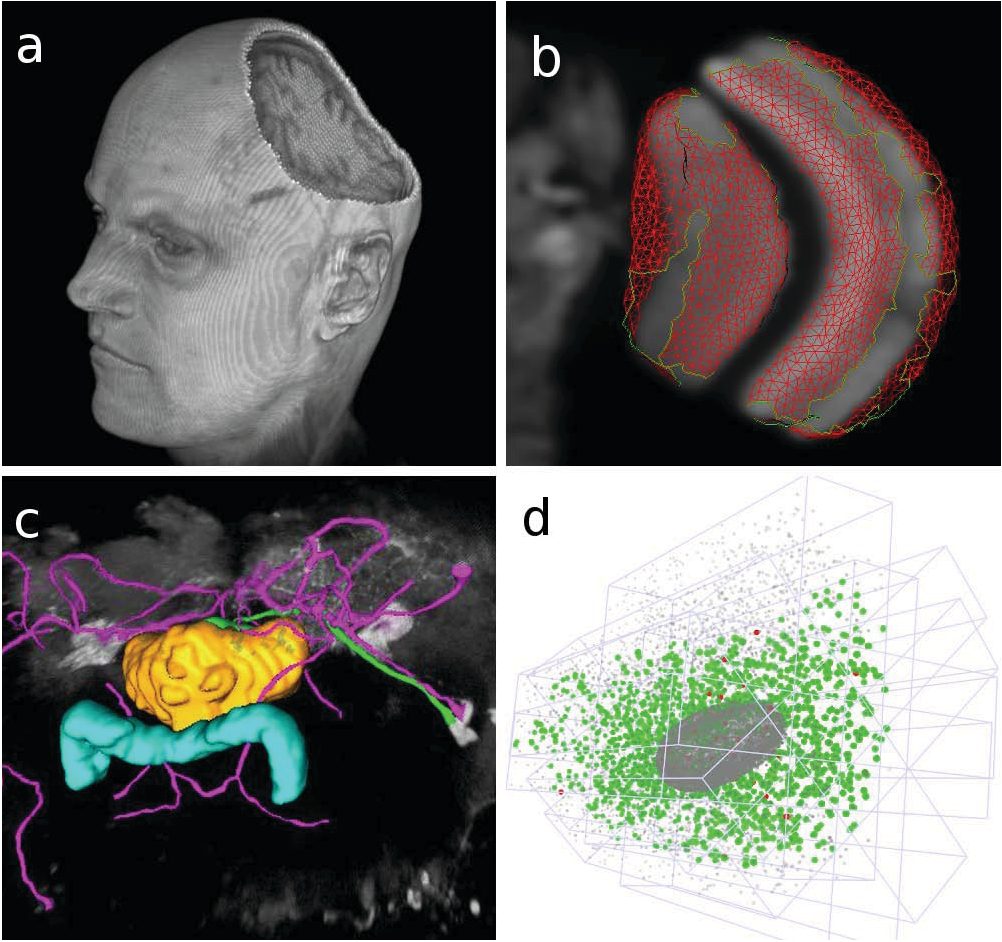
**Example applications**. (a) An MRI image of a human head, demonstrating the volume editing capabilities: A 2D ROI was projected onto the data, and the intersected volume was filled with black. Afterwards the head was rotated to show the effect. (b) Visualization of the output of a segmentation algorithm. The segmented image is a confocal stack of an adult *Drosophila *brain. Shown here is the right optic lobe. The segmentation surface (in *red*) resembles the boundaries of the medulla and lobula. (c) Snapshot of the Simple Neurite Tracer application, featuring the central compartments of the adult *Drosophila *brain. The intensity image is displayed as a volume rendering (*gray*). The protocerebral bridge and the fan-shaped body are shown as surface renderings (*cyan *&*yellow*). The traced neural tracts are displayed as custom meshes (*magenta *&*green*). (d) Visualization of multiple view registration computed from correspondences of fluorescent beads. Multiple views were obtained by SPIM imaging of a *Drosophila *embryo. Beads are rendered as point meshes.

Additionally, volume editing is possible interactively: The representation of an image stack in a 3D window enables 2D regions of interest to be projected across arbitrary axes of the volume. This enables what we refer to as "3D cropping", which consists of setting all voxels in the image volume that fall within the projected 2D region of interest to a particular color, typically black. We use 3D cropping to remove arbitrary parts of an image volume to inspect regions deep into the volume (Figure 5a).

#### Annotation in 3D space

The 3D scene can display landmark annotations for each image volume. These are added using the point tool. Existing landmarks are listed in a table that allows the manipulation of their properties, such as name and color. Each image volume hosted in the 3D scene may have an associated set of 3D landmarks of this type. A set of landmarks may be stored in a file for analysis, and reloaded in subsequent annotation sessions.

#### Landmark-based 3D rigid registration of image volumes

Two sets of homonymous landmarks positioned over two corresponding image volumes can be used for estimating a rigid transformation model [11] (see also Additional file 1, Figure S1). Using this model, one image volume can be aligned onto the other. The "Transform" menu offers options for exporting the transformed image volume as an image stack suitable for further processing with ImageJ.

#### Animation and recording

The 3D viewer offers an option to record a 360-degree rotation of any 3D scene. Additionally, a recording mode is available. When this is activated, every manual rotation, translation and zooming of the display or any of its elements is recorded; when stopped, the recording is displayed as an ImageJ stack. Recordings may be output as videos via ImageJ.

#### Custom content

Beyond the three basic image volume display types (volume rendering, mesh and or-thoslice set), the 3D scene accepts custom-crafted meshes. These meshes are typically generated programmatically, such as by automatic segmentation of image stacks.

#### 4D Visualization

Time-lapse recordings of 3D data sets can be loaded and visualized in the 3D scene. Standard command buttons for play, pause, fast-forward, etc. control the time point displayed in the viewer. Interactive zooming, rotation and panning are enabled as the time sequence progresses. When paused, the visualization of the current time point may be annotated, interacted with and measured as with any other 3D scene.

### Usage as a GUI application

Our 3D visualization library includes a fully-functional plugin for ImageJ named "3D Viewer". The plugin is listed automatically in ImageJ's plugin menus. When executed, the plugin creates a new 3D scene, and automatically offers a dialog for displaying any open image stack as an image volume. The dialog provides the means to alter the attributes of the image volume, such as its representation type (volume rendering, isosurface (mesh) or orthoslices), and its color and transparency settings. The menu of the 3D scene window offers options for inserting further image volumes and editing, annotating and transforming them. Extensive documentation is available online http://3dviewer.neurofly.de, along with video tutorials and a 'Frequently Asked Questions' section.

### Usage as a programming library

Our framework exposes a public API to allow applications to integrate its features. A basic example demonstrates the use-case of visualizing in 3D an image volume and a mesh (see below). The example illustrates the development of an image segmentation algorithm, which extracts the boundary of the structures of interest as surfaces and represents them as a mesh composed of triangles. First, the image volume is rendered as orthoslices. Then the surface is displayed.

The first step is to instantiate an object of the class Image3DUniverse. Then we call its show() method, which creates a window to interact with the 3D scene. The scene graph is setup automatically.

Image3DUniverse univ = new Image3DUniverse(640, 480);

univ.show();

Next, the image volume is loaded. We display it as orthoslices in the 3D scene by calling the addOrthoslice() method:

ImagePlus imp = IJ.openImage("flybrain.tif");

Content c = univ.addOrthoslice(imp);

Alternatively, instead of addOrthoslice(), addVoltex() or addMesh() could be used to display the image as a volume or isosurface rendering, respectively.

If we assume that there exists an external method createVertices() that creates a list of points describing the vertices of the surface, and that three consecutive vertices define a triangle, the following source code shows how to create a custom triangle mesh and add it to the scene:

List<Point3f> vertices = createVertices();

CustomMesh cm = new CustomTriangleMesh(vertices);

univ.addCustomMesh(cm, "triangle mesh");

The result looks similar to Figure 5b, which shows a confocal image of a fly brain together with parts of the surface of the medulla and the lobula (two compartments of the optic lobe).

Another API example illustrates a simulation of dendritic growth (Figure 4 and Additional file 1, section 2). The source code uses direct edits of the volumetric data to represent the growth over time. Documentation in the form of source code examples is available online at http://3dviewer.neurofly.de, in the *Developer HowTos *category. The documentation demonstrates in a tutorial style the available functionality of our framework.

## Discussion

Numerous ImageJ-based applications currently use our 3D visualization library. We briefly discuss below how three key applications use our library, illustrating the breadth of functionality we provide. We then conclude with future perspectives considering new demands for image processing and visualization in biomedical research.

The Simple Neurite Tracer [12] is an ImageJ plugin for semi-automated tracing of neurons in 3D image data. The application provides semiautomatic segmentation of filament-like structures such as neural arborizations and blood vessels. A starting point is chosen and then the filament is auto-traced up to a desired end point. The traced 3D path is visualized using components of our framework (Figure 5c). This example demonstrates how an analytical tool for measuring complex 3D structures can be augmented with 3D visualization capabilities to display those objects.

An algorithm has been developed for registering images of a 3D sample, where each image volume represents a different angle of view obtained by Single Plane Illumination Microscopy [13]. The implementation of this complex algorithm required the 3D visualization of intermediate and final image registration steps. Our library enabled the algorithm developers to generate the required visualizations with very little effort (Figure 5d).

TrakEM2 is an ImageJ plugin for visualization, analysis, segmentation, reconstruction and registration of very large 3D image data sets obtained by serial section electron microscopy [14]. TrakEM2 makes extensive usage of our framework for interaction with the 3D representation of image volumes and segmented objects of interest. The development of our library empowered TrakEM2 developers to plan and design for 3D interactive features that would not have been possible otherwise. Reciprocally, the high-performance requirements of TrakEM2 drove implementation of parallel processing strategies for isosurface extraction and mesh composition in the 3D scene.

The interaction of our library with other software packages, each with specific requirements, promotes the development of new features and improves performance. These improvements then propagate back and enhance other ImageJ applications. Detailed information about the use of the program and downloadable example code are available on our web page at http://3dviewer.neurofly.de.

The advent of high-throughput microscopy has increased the number and size of biological image data sets in need of analysis. The acquisition of 4D data, such as from laser-scanning fluorescent microscopy of cells moving through space, has become commonplace. Interactive data analysis of 4D data sets for object motion tracking is in increasing demand. Our framework contains all the key ingredients for 4D visualization and 4D data representation. For the near future, we will target the addition of convenient analytical tools that consider the time dimension. The ease of use and open source nature of our library enables the development of custom solutions for the highly specialized needs of biomedical image analysis.

## Conclusions

In this paper, we introduced a new high-level 3D visualization framework for ImageJ. The framework provides an interactive 3D scene for image volume visualization, annotation, segmentation and transformation. For programmers, it offers the means to trivially augment the capabilities of their custom applications with hardware-accelerated 3D visualization. The framework has been very well received by the ImageJ user and developer community, and is currently in use by numerous ImageJ-based applications.

## Availability and requirements

**Project name**: ImageJ 3D Viewer

**Project home page**: http://3dviewer.neurofly.de

**Operating systems(s)**: Platform independent

**Programming language**: Java and Java 3D

**Other requirements**: ImageJ

**Any restrictions to use by non-academics**: none

A JAR archive containing the software (with source code) can be downloaded from the project home page, following the *Download *link. Java 3D is available from https://java3d.dev.java.net and ImageJ from http://rsbweb.nih.gov/ij. The easiest way to set up these components is to install Fiji http://pacific.mpi-cbg.de, which bundles these dependencies and the software we present here. Additionally, a movie demonstrating its basic usage is provided as Additional file 3, and the software in its current state as Additional file 4.

## Authors' contributions

BS designed and wrote the main body of the library. JS implemented numerous algorithms such as for image transformation and registration. AC implemented the parallelization of image volume processing and improved the graphical user interface. ML implemented segmentation algorithms and worked on the public API. MH identified the need for the library and coordinated its implementation. All authors read and approved the final manuscript.

## Supplementary Material

Additional file 1 **Supplementary material**. *Top*, demonstration of landmark selection in two different adult *Drosophila *brains, for the purpose of landmark-based image volume registration. *Bottom*, source code example implementing the dendritic growth simulation shown in Figure 4 of the main manuscript. Click here for file

Additional file 2 **Direct volume editing**. A movie which shows the result of the simulated dendritic growth. The corresponding source code is presented in Additional file 1, section 2. Click here for file

Additional file 3 **Basic usage**. A movie which demonstrates the basic usage of the ImageJ plugin provided by our framework. More screen casts are available on our web page. Click here for file

Additional file 4 **Software**. The JAR archive, containing both binary classes and the Java source code of our software. To install the software, this file must be copied into ImageJ's plugins directory. Click here for file

## References

[B1] Clendenon JL Voxx: a PC-based, near real-time volume rendering system for biological microscopy Am J Physiol Cell Physiol 2002 282 C213 C218 11742814 10.1152/ajpcell.2002.282.1.C213

[B2] Peng H V3D 2009 http://penglab.janelia.org/proj/v3d/v3d2.html

[B3] Pettersen EF Goddard TD Huang CC Couch GS Greenblatt DM Meng EC Ferrin TE UCSF Chimera-a visualization system for exploratory research and analysis Journal of computational chemistry 2004 25 13 1605 1612 10.1002/jcc.20084 15264254

[B4] Abràmoff M VolumeJ 2003 http://bij.isi.uu.nl/index.htm

[B5] Barthel KU Volume Viewer 2005 http://rsb.info.nih.gov/ij/plugins/volume-viewer.html

[B6] Schindelin J Fiji is just ImageJ - Batteries included Proceedings of the ImageJ User and Developer Conference, Luxembourg 2008

[B7] MessaoudiI C Boudier T Sorzano C Marco S TomoJ: tomography software for three-dimensional reconstruction in transmission electron microscopy BMC Bioinformatics 2007 8 288 10.1186/1471-2105-8-288 17683598 PMC1976622

[B8] Sun Microsystems Java 3D Engineering Team Java 3D API Tutorial 2000 http://java.sun.com/developer/onlineTraining/java3d

[B9] Gehringer D Java 3D Volume Rendering 2006

[B10] Lorensen WE Cline HE Marching cubes: A high resolution 3D surface construction algorithm SIGGRAPH '87: Proceedings of the 14th annual conference on Computer graphics and interactive techniques 21 New York, NY, USA: ACM Press 163 169

[B11] Horn BK Closed-form solution of absolute orientation using unit quaternions Journal of the Optical Society of America 1987 4 624+

[B12] Longair M Computational Neuroanatomy of the Central Complex of Drosophila melanogaster PhD thesis 2009 University of Edinburgh, School of Informatics http://homepages.inf.ed.ac.uk/s9808248/imagej/tracer

[B13] Preibisch S Saalfeld S Rohlfing T Tomancak P Pluim JPW, Dawant BM Bead-based mosaicing of single plane illumination microscopy images using geometric local descriptor matching Medical Imaging 2009: Image Processing 2009 7259 SPIE 72592S

[B14] Cardona A Saalfeld S Tomančák P Hartenstein V Drosophila brain development: closing the gap between a macroarchitectural and a microarchitectural approach Cold Spring Harb Symp Quant Biol 2009 sqb.2009.74.037 epub 20028843 10.1101/sqb.2009.74.037 PMC3950651

