# Supplementary material for: A high-level 3D visualization API for Java and ImageJ
Source: BMC Bioinformatics. 2010 May 21;11:274. doi: 10.1186/1471-2105-11-274 (PMC2896381; doi:10.1186/1471-2105-11-274)
Supplement: Additional file 1 — Supplementary material. Top, demonstration of landmark selection in two different adult Drosophila brains, for the purpose of landmark-based image volume registration. Bottom, source code example implementing the dendritic growth simulation shown in Figure 4 of the main manuscript. [file 1471-2105-11-274-S1.PDF]

Supplementary Data

1 Example images

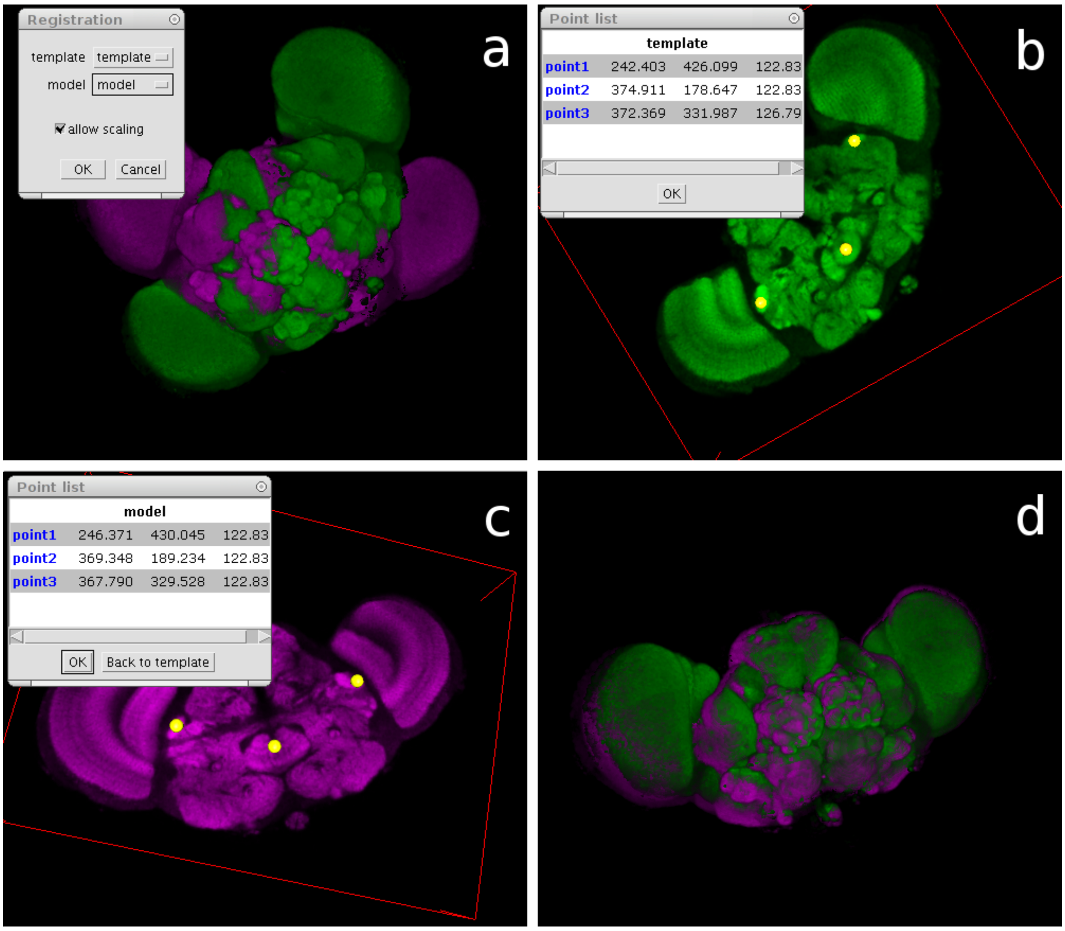

Figure 1: Registration. (a) Two unaligned *Drosophila* brains. The reference brain is shown in green, the model brain (the one to be registered) is shown in magenta. (b) Landmark selection in the model brain. The display mode is automatically changed to orthoslices. The user can scroll through the image planes and select landmarks, which are displayed in a separate dialog window. Landmarks are shown yellow in the figure. They can be moved, highlighted and renamed. (c) Subsequent landmark selection of the template brain. (d) The landmarks are used to infer a rigid transformation which aligns the model image to the landmark image.

2 A more advanced API example simulating dendritic growth in the thorax of a *Drosophila*

We designed our framework with a dual purpose. On the one hand, to provide an interactive 3D scene for end-users. On the other hand, to provide an easy programming interface to instantiate a 3D scene and manipulate its internals. Programmers can trivially augment applications with 3D visualization capabilities. This section demonstrates the latter aspect. A working example is listed. Similar examples and tutorials, together with source code, are found online at `http://3dviewer.neurofly.de`.

```java
import ij3d.Image3DUniverse;
import ij3d.Content;

import voltex.VoltexVolume;
import voltex.VoltexGroup;

import ij.IJ;
import ij.ImageJ;
import ij.ImagePlus;

import java.io.DataInputStream;
import java.io.FileInputStream;
import java.io.EOFException;

public class Editable_Volume {

    public static void main(String[] args) throws Exception {
        new ImageJ();

        // Create a 3D scene and show it
        Image3DUniverse univ = new Image3DUniverse(600, 480);
        univ.show();

        // Open the image and add it as a volume
        ImagePlus image = IJ.openImage("thorax.tif");
        Content c = univ.addVoltex(image);
        c.setTransparency(0.7f);

        // Adjust the view
        univ.rotateToNegativeXY();
        univ.waitForNextFrame();
        univ.getViewPlatformTransformer().zoom(40);

        // Retrieve the VoltexVolume of this volume rendering
        VoltexVolume volume = ((VoltexGroup)c.getContent()).
                    getRenderer().getVolume();

        // Read in data from file
        DataInputStream is = new DataInputStream(
                    new FileInputStream("thorax.stream"));

        while (true) {
            try {
                int x = is.readInt();
                int y = is.readInt();
                int z = is.readInt();
                int v = is.readInt();
                volume.set(x, y, z, v);
            } catch(EOFException ex) {
                break;
            }
        }
        is.close();
    }
}
```

Listing 1: The source code here opens an image, renders it as a volume rendering in a virtual universe and subsequently changes voxel values, as read from a stream.

Listing 1 shows how an instance of `Image3DUniverse` is created. An image is opened and added to the universe as a volume rendering via `addVoltex()`. This method returns the new `Content`, which is then used to make it partially transparent.

After adjusting the view, the `VoltexVolume` of the volume rendering is retrieved. The `VoltexVolume` allows to change voxel values in volume renderings directly. The example shows how the values are read from a file input stream, which are interpreted as tuples of 4

consecutive integer values, specifying the x, y and z coordinate and the new voxel value.

The example uses data obtained via a segmentation algorithm. The red channel shows an antibody staining, visualizing the expression pattern of a *Drosophila* GAL4 driver line. This channel was used to simulate axon growth.

The full source code can be found at `http://3dviewer.neurofly.de/?page=bioinf09/source&category=Publications`. Next to the source code, it explains the requirements, how to compile and how to run the example. Additionally, it provides a Java webstart application which offers to run the example with one click (i.e. without requiring installation), and a demo movie.
