# Supplementary figures and images for: A high-level 3D visualization API for Java and ImageJ
Source: BMC Bioinformatics. 2010 May 21;11:274. doi: 10.1186/1471-2105-11-274 (PMC2896381; doi:10.1186/1471-2105-11-274)

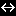

Supplement: Additional file 4 — Software. The JAR archive, containing both binary classes and the Java source code of our software. To install the software, this file must be copied into ImageJ's plugins directory. [file 1471-2105-11-274-S4.ZIP › view4d/icons/bounceback.png]

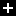

Supplement: Additional file 4 — Software. The JAR archive, containing both binary classes and the Java source code of our software. To install the software, this file must be copied into ImageJ's plugins directory. [file 1471-2105-11-274-S4.ZIP › view4d/icons/faster.png]

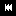

Supplement: Additional file 4 — Software. The JAR archive, containing both binary classes and the Java source code of our software. To install the software, this file must be copied into ImageJ's plugins directory. [file 1471-2105-11-274-S4.ZIP › view4d/icons/first.png]

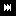

Supplement: Additional file 4 — Software. The JAR archive, containing both binary classes and the Java source code of our software. To install the software, this file must be copied into ImageJ's plugins directory. [file 1471-2105-11-274-S4.ZIP › view4d/icons/last.png]

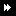

Supplement: Additional file 4 — Software. The JAR archive, containing both binary classes and the Java source code of our software. To install the software, this file must be copied into ImageJ's plugins directory. [file 1471-2105-11-274-S4.ZIP › view4d/icons/next.png]

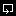

Supplement: Additional file 4 — Software. The JAR archive, containing both binary classes and the Java source code of our software. To install the software, this file must be copied into ImageJ's plugins directory. [file 1471-2105-11-274-S4.ZIP › view4d/icons/nobounceback.png]

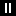

Supplement: Additional file 4 — Software. The JAR archive, containing both binary classes and the Java source code of our software. To install the software, this file must be copied into ImageJ's plugins directory. [file 1471-2105-11-274-S4.ZIP › view4d/icons/pause.png]

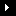

Supplement: Additional file 4 — Software. The JAR archive, containing both binary classes and the Java source code of our software. To install the software, this file must be copied into ImageJ's plugins directory. [file 1471-2105-11-274-S4.ZIP › view4d/icons/play.png]

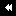

Supplement: Additional file 4 — Software. The JAR archive, containing both binary classes and the Java source code of our software. To install the software, this file must be copied into ImageJ's plugins directory. [file 1471-2105-11-274-S4.ZIP › view4d/icons/previous.png]

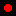

Supplement: Additional file 4 — Software. The JAR archive, containing both binary classes and the Java source code of our software. To install the software, this file must be copied into ImageJ's plugins directory. [file 1471-2105-11-274-S4.ZIP › view4d/icons/record.png]

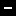

Supplement: Additional file 4 — Software. The JAR archive, containing both binary classes and the Java source code of our software. To install the software, this file must be copied into ImageJ's plugins directory. [file 1471-2105-11-274-S4.ZIP › view4d/icons/slower.png]

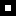

Supplement: Additional file 4 — Software. The JAR archive, containing both binary classes and the Java source code of our software. To install the software, this file must be copied into ImageJ's plugins directory. [file 1471-2105-11-274-S4.ZIP › view4d/icons/stop.png]
